# Characterization of *Arabidopsis thaliana* R2R3 S23 MYB Transcription Factors as Novel Targets of the Ubiquitin Proteasome-Pathway and Regulators of Salt Stress and Abscisic Acid Response

**DOI:** 10.3389/fpls.2021.629208

**Published:** 2021-08-19

**Authors:** Chase Beathard, Sutton Mooney, Raed Al-Saharin, Aymeric Goyer, Hanjo Hellmann

**Affiliations:** ^1^School of Biological Sciences, Washington State University, Pullman, WA, United States; ^2^Department of Applied Biology, Tafila Technical University, At-Tafilah, Jordan; ^3^Department of Botany and Plant Pathology, Hermiston Agricultural Research and Extension Center, Oregon State University, Hermiston, OR, United States

**Keywords:** MYB, transcription factor, E3 ligase, BPM, abiotic stress, CRL3, CUL3, S23

## Abstract

Rapid response to environmental changes and abiotic stress to coordinate developmental programs is critical for plants. To accomplish this, plants use the ubiquitin proteasome pathway as a flexible and efficient mechanism to control protein stability and to direct cellular reactions. Here, we show that all three members of the R2R3 S23 MYB transcription factor subfamily, MYB1, MYB25, and MYB109, are degraded by the 26S proteasome, likely facilitated by a CUL3-based E3 ligase that uses MATH-BTB/POZ proteins as substrate adaptors. A detailed description of *MYB1*, *MYB25*, and *MYB109* expression shows their nuclear localization and specific tissue specific expression patterns. It further demonstrates that elevated expression of MYB25 reduces sensitivities toward abscisic acid, osmotic and salt stress in Arabidopsis, while downregulation of all S23 members results in hypersensitivities. Transcriptional profiling in root and shoot of seedlings overexpressing MYB25 shows that the transcription factor widely affects cellular stress pathways related to biotic and abiotic stress control. Overall, the work extends our knowledge on proteins targeted by CUL3-based E3 ligases that use MATH-BTB/POZ proteins as substrate adaptors and provides first information on all members of the MYB S23 subfamily.

## Introduction

The control of protein stability is a central tool for plants to quickly respond to environmental changes, and to efficiently coordinate developmental and physiological processes. A key regulatory mechanism to accomplish this is the ubiquitin proteasome pathway, which marks selected proteins with ubiquitins (UBQ) (Choi et al., 2014). This process utilizes an E3 ligase to bind with a substrate, facilitating its ubiquitylation. Classically, building up an UBQ chain results in the proteolytic degradation of the marked protein by the 26S proteasome (Choi et al., 2014).

One class of E3 ligases that consists of a CULLIN 3 (CUL3), a RING-finger protein RBX1, and a MATH-BTB/POZ (BPM) protein (further denoted as CRL3^BPM^) appears to be involved predominantly in the control of transcriptional processes (Weber and Hellmann, 2009; Lechner et al., 2011; Juranic et al., 2012; Chen et al., 2013, 2015; Ma et al., 2015; Morimoto et al., 2017). While the cullin serves as a central scaffolding subunit, RBX1 binds an E2/UBQ complex, and the BPM moiety serves as a substrate adapter (Weber et al., 2005; Weber and Hellmann, 2009). Arabidopsis expresses six BPM proteins and so far, all identified substrates have been transcription factors, and include members from four major families: R2R3 myeloblastosis (MYB), APETALA2/ERF binding factors (AP2/ERF), myelocytoma (MYC) and class I homeobox-leucine zipper (HB) (Weber et al., 2005; Weber and Hellmann, 2009; Lechner et al., 2011; Chen et al., 2013, 2015; Mooney et al., 2019; Chico et al., 2020).

In Arabidopsis, the R2R3-MYB superfamily has 126 members that belong to 24 different subfamilies. Amongst those, MYB56, a member of the closely related S21 subfamily, is a target of CRL3^BPM^ activities (Chen et al., 2015). MYB56 has been described to be involved in brassinosteroid signaling, embryo size, anthocyanin biosynthesis, and flowering time (Zhang et al., 2013; Vilarrasa-Blasi et al., 2014; Chen et al., 2015; Jeong et al., 2018). Most of these conclusions were reached by phenotypic analysis of plants overexpressing *MYB56*, with only minor phenotypical changes being observed in loss-of-function mutants, potentially due to functional redundancy within the S21 subfamily (Stracke et al., 2001; Chen et al., 2015).

Based on our previous work that showed MYB56 to be a novel MYB-family target, we were interested in how broadly the CUL3-BPM ligase interacted with the MYB-super family. In general, MYB transcription factors have been shown to play important roles across all facets of development and response. Understanding the interplay of these factors may reveal novel ways to harness the UBQ-proteasome system to increase the ability of crops to cope with environmental stressors that impact yield. Because the S23 R2R3-MYB subfamily consists of three proteins, MYB1, MYB25, and MYB109, for which a biological role has not been identified yet, we chose these as novel candidates to investigate. Here we describe that all members of this subfamily are 26S proteasome targets and interact with BPM proteins. We also show the tissue expression pattern of MYB1, MYB25 and MYB109 by GUS-protein fusion analysis. In addition, we demonstrate that MYB25 overexpression in Arabidopsis alters salt stress and ABA sensitivities, and that transcriptome changes in MYB25-overexpressing plants are associated with various abiotic and biotic pathways, suggesting a role in plant stress response.

## Results

### Structural Organization of the R2R3 S23 Subfamily

We previously described that members of the S21 MYB transcription factor subfamily are targets of a CUL3-based E3 ligase that uses BPM proteins as substrate adaptors (Chen et al., 2015). In anticipation that these E3 ligases can potentially target additional MYB transcription factors outside the S21 subfamily, we analyzed the S23 subfamily, which includes three members: MYB1 (At3g09230; 393 AA; 42.81 kDa), MYB25 (At2g39880; 367 AA; 40.91 kDa), and MYB109 (At3g55730; 399 AA; 43.53 kDa) (Stracke et al., 2001). Homology and similarity comparisons by us (Supplementary Figure 1), and phylogenetic analysis done by Stracke et al. (2001) has shown that MYB25 and MYB109 group most closely together, while MYB1 forms a separate clade. It needs to be mentioned here that SALT-RELATED MYB1 (SRM1/MYBS2/At5g08520) (Wang et al., 2015), despite its listing on TAIR as MYB1^1^, is not a member of the S23 subfamily, and has not been assigned to the R2R3 family (Dubos et al., 2010).

**FIGURE 1:**
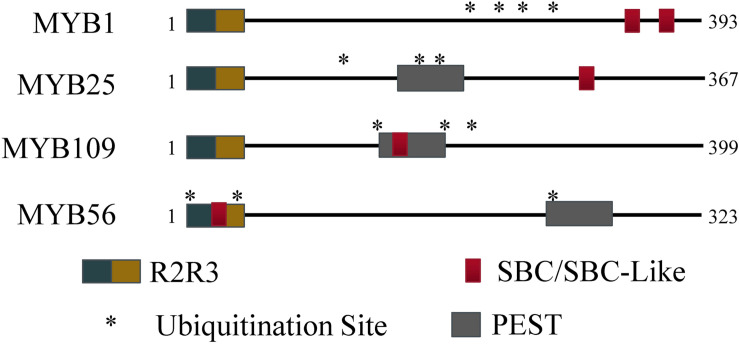
Protein regions for MYB56 and the S23 subfamily. All S23 members have an R2R3 domain at the N-terminus. Predicted SBC and PEST regions are present to varying degrees among the S23 subfamily, however all three members contain at least three predicted ubiquitination residues (indicated by *) and at least one SBC or PEST motif.

The R2R3 S23 MYB transcription factor subfamily contains several known motifs. Using the ELM server tool^2^, R2R3 DNA binding motifs were identified in the N-terminal regions (MYB1: 54–103 and 106–154; MYB25: 49–98 and 101–146; MYB109: 55–104 and 107–155). In addition, PEST motifs were predicted by the epestfind tool^3^ for MYB25 (AA 186–200) and MYB109 (AA 177–191), but not for MYB1 (Figure 1). PEST motifs are enriched in the amino acids proline (P), glutamate (E), serine (S), and threonine (T), and are often connected with protein instability (Salama et al., 1994; Tsurumi et al., 1995; Berset et al., 2002). They have been demonstrated in some BPM substrates to be critical for BPM-substrate interaction (Mooney et al., 2019).

In humans and plants, a conserved SPOP-binding Consensus (SBC) motif was identified that facilitates interaction between substrate and BPM proteins (Zhuang et al., 2009; Morimoto et al., 2017). In humans, the cassette is often enriched in serine and threonine residues, and generally follows the sequence motif ϕ-π-S-S/T-S/T (ϕ, non-polar; π, polar; S, serine; T, threonine), while in plants a slightly modified version is also recognized by BPMs (ϕ-π-S-X-S/T, with X being any amino acid). Queries of the S23 MYB sequences revealed that MYB1 contains an SBC (LESSS, AA 350–355) and an SBC-like (LDSPT, AA 323–328) motif, while MYB25 only contains an SBC (LSSSS; AA 282–287) and MYB109 an SBC-like (ANSVT; AA 167–172) motif (Figure 1). The presence of SBC and/or PEST motifs indicates that all three MYB proteins may interact with BPM proteins and be targets of the ubiquitin-proteasome pathway, respectively. In agreement with this, in all three cases, the UbPred tool^4^ predicted several lysine residues that have a high confidence level to be ubiquitylation sites (Figure 1). MYB56–a previously confirmed CRL3^BPM^ substrate (Chen et al., 2015)–also contains PEST and SBC-like motifs as well as ubiquitylation sites (Figure 1). Because of these similarities, we decided to further verify whether the S23 MYB proteins are BPM substrates.

### The R2R3 S23 Subfamily Interacts With BPM Proteins in Yeast and *in vitro*

**FIGURE 2:**
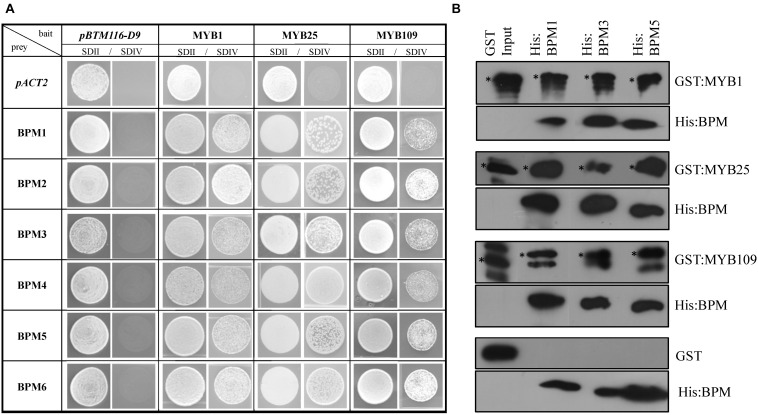
Yeast 2-hybrid and *in vitro* pulldown assays show interaction of S23 family members with the Arabidopsis BPM family. **(A)** Yeast 2-hybrid analysis shows that MYB1, MYB25, and MYB109 can interact with all members of the Arabidopsis BPM family. SDII is the selection medium for transformation with the bait vector *pBTM112-D9*, and the prey plasmid *pACT2* supplemented with uracil and His; SDIV is the selection medium for interaction studies without uracil and His supplements. Images were taken from single spots. **(B)** Pulldown analysis with purified, recombinant proteins show that all three His-tagged BPM proteins (His:BPM1, :BPM3, and :BPM5) were able to co-precipitate GST-tagged MYB1, MYB25, and MYB109, while GST alone did not interact with any of the His:BPM proteins. If not otherwise stated, all results in this and subsequent figures were repeated at least three times independently. ^∗^, indicate full-length recombinant GST-fusion proteins (GST:MYB1 ∼69.7 kDa; GST:MYB25 ∼67.8; GST:MYB109 ∼70.4 kDa). Additional bands are likely either unspecific cross-reactions with the antibody or truncated recombinant protein.

To test interaction between the S23 subfamily members and BPMs, we followed two approaches. Yeast 2-hybrid (Y2H) assays were performed between the three S23 MYBs and all Arabidopsis BPMs. As shown in Figure 2A, all of the BPMs are able to interact with MYB1, MYB25, and MYB109. To further corroborate these findings, pulldown assays were performed with three His-tagged BPM proteins that represent members from two of the three BPM clades present in Arabidopsis (BPM1, 3, and 5) (Weber et al., 2005), and GST-tagged MYB proteins. While GST:MYB1, :MYB25, and :MYB109 were all able to co-precipitate with the different His:BPMs, GST alone was not (Figure 2B). These results provide evidence that MYB1, MYB25, and MYB109 can be substrates of a CUL3^BPM^ E3 ligase.

### S23 MYB Proteins Are Unstable in a Proteasome-Dependent Manner

**FIGURE 3:**
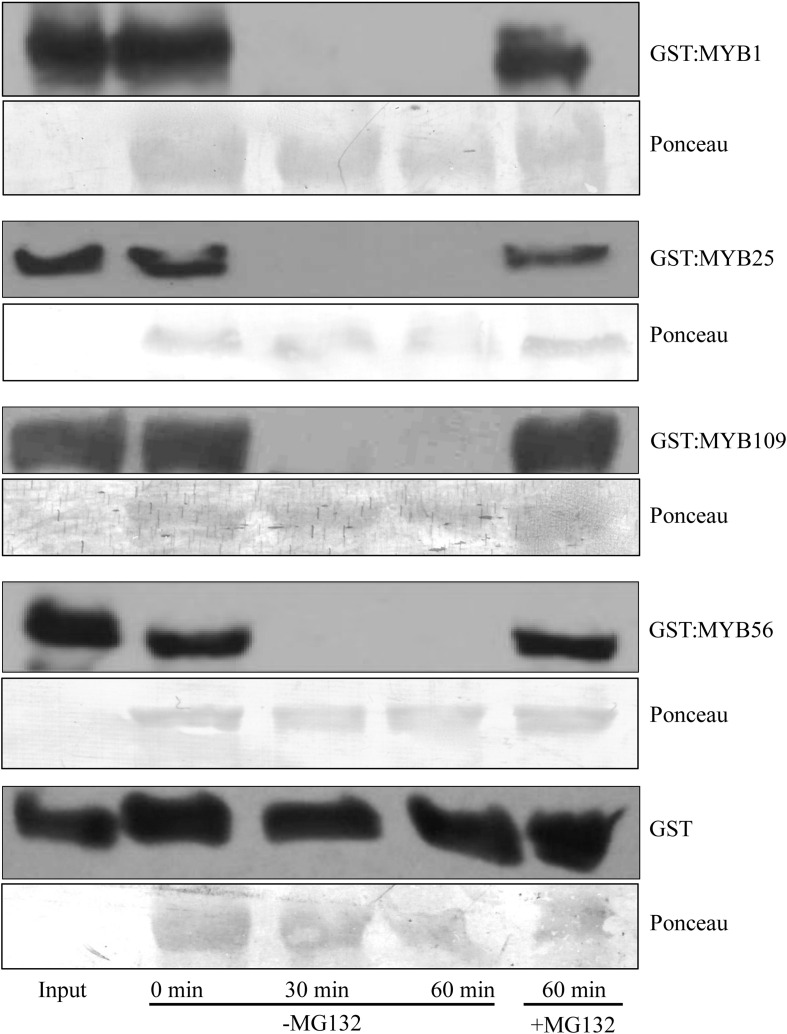
Cell-free degradation assays show 26S proteasome-dependent instability of the S23 subfamily. Purified GST:MYB1, :MYB25, and :MYB109 proteins become fully degraded in cell-free degradation assays within 30 min. MG132, a proteasomal inhibitor, stabilizes the MYB proteins, demonstrating that their degradation requires activity of the 26S proteasome. GST was used as a negative control and remained stable throughout the incubation period of up to 60 min. Ponceau staining was used as loading control. The visible bands shown correlate in size with the large subunit of Rubisco (∼55 kDa).

To verify whether the S23 subfamily members are substrates of the 26S proteasome, we utilized a cell-free degradation assay that contains all the necessary components to degrade a protein through the ubiquitin proteasome pathway (Wang et al., 2009; Mooney et al., 2019). Purified, recombinant S23 proteins tagged with a GST incubated in native plant extracts and tracked over a 60 min time course to determine if their amount decreased over time (Figure 3). As controls we included GST:MYB56 and GST alone. In addition, the proteasomal inhibitor MG132 was used to test whether degradation depends on proteasomal activity. GST:MYB1, :MYB25, :MYB109, and :MYB56 were entirely degraded after 30 min, and in all cases degradation was inhibited in the presence of the proteasomal inhibitor MG132. In comparison, GST alone, which served as a negative control, was fully stable over the tested time range showing that degradation of the recombinant proteins was MYB-dependent. Overall, these results indicate that all members of the S23 subfamily are substrates of the 26S proteasome.

### Tissue Specific Expression and Subcellular Localization of MYB1, MYB25, and MYB109

Since no detailed functional descriptions of S23 family members are available, we analyzed MYB1, MYB25, and MYB109 to determine expression patterns in tissue and intracellular locations.

**FIGURE 4:**
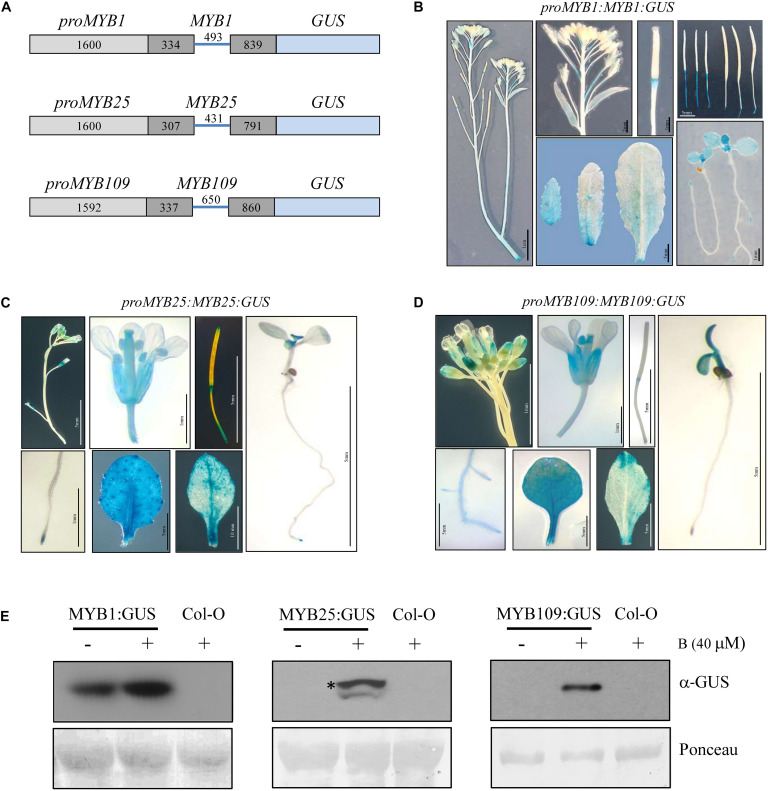
Tissue specific expression pattern and stability of MYB1, MYB25, and MYB109. **(A)** Schematic drawings of MYB1, MYB25, and MYB109 genomic expression constructs. For all *MYB* constructs length of the cloned promoters and introns and exons are given in base pairs. Light gray bars represent promoter regions, dark gray bars represent exons, blue lines in between represent an intron. To accomplish translational fusions with GUS, the stop codons of *MYB1*, *MYB25*, and *MYB109* coding regions were deleted. GUS expression patterns in plants carrying either *proMYB1:MYB1:GUS* **(B)**, *proMYB25:MYB25:GUS* **(C)**, or *proMYB109:MYB109:GUS* **(D)** expression constructs. **(E)** α-GUS Western blot analysis shows that overnight treatment with the proteasomal inhibitor bortezomib (B) leads to increased MYB1:GUS, MYB25:GUS, and MYB109:GUS proteins. Ponceau staining was used as loading control. The visible bands shown correlate in size with the large subunit of Rubisco (∼55 kDa). * indicates full-length MYB25:GUS protein.

Tissue specific expression patterns for *MYB1*, *MYB25*, and *MYB109* were investigated by generating transgenic plants that expressed a *GUS* reporter under the control of a genomic construct that comprised the promoter (*MYB1* and *MYB25*, 1600 bp upstream of the ATG; *MYB109*, 1592 bp upstream of the ATG) and the entire gene without the 3′ untranslated region (further referred to as *proMYB1:MYB1:GUS*, *proMYB25:MYB25:GUS*, and *proMYB109:MYB109:GUS*, respectively) (Figure 4A). Several independent transgenic lines were obtained for each construct and analyzed for GUS activity. Plants expressing the *proMYB1:MYB1:GUS* construct mainly showed GUS expression in developing leaves (cotyledons and young rosette leaves) and in the root tips (Figure 4B). Staining was not observable in stem, flowers or seeds, while there was strong staining at the pedicels connecting the flowers and siliques with the main stem (Figure 4B). Generally, expression of the reporter among the *proMYB25:MYB25:GUS* and *proMYB109:MYB109:GUS* constructs was very comparable (Figures 4C,D). The corresponding plants showed GUS expression in roots, young leaves, anthers, and the extremities of siliques, and expression patterns for the MYB25 and MYB109 expression constructs were highly comparable (Figures 4C,D).

While GUS protein was detectable in proMYB1:MYB1:GUS expressing plants, it was not detectable in the *proMYB25:MYB25:GUS* and *proMYB109:MYB109:GUS* lines. Figure 4E indicating that MYB25:GUS and MYB109:GUS are highly unstable, possibly due to degradation by the proteasome. Supporting this notion, GUS protein was detected (single band of around 100 kDa) in *proMYB25:MYB25:GUS* and *proMYB109:MYB109:GUS* plants treated with the proteasomal inhibitor bortezomib for 16 h, while no protein was detectable for bortezomib-treated wild type plants on Western blot (Figure 4D). We did observe increased protein amounts MYB1:GUS after plants were treated with bortezomib (Figure 4E). These data further corroborated findings from the cell-free degradation assays and provide strong evidence that all three S23 members are targets of the 26S proteasome *in planta*.

**FIGURE 5:**
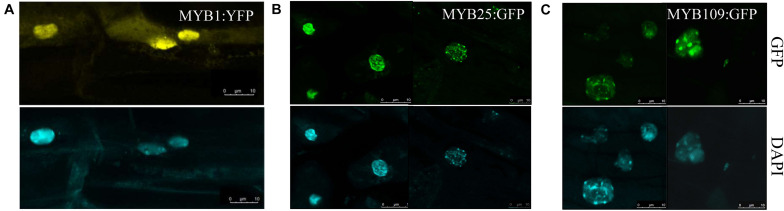
MYB1, MYB25, and MYB109 are localized in the nucleus. Expression analysis in transgenic *Arabidopsis thaliana* showed that YFP-tagged MYB1 **(A)**, or GFP-tagged MYB25 **(B)** and MYB109 **(C)** proteins are localized in the nucleus. 4′,6-diamidino-2-phenylindole (DAPI) images are included to indicate the location of the nuclei.

Subcellular localization studies were done with either a YFP-reporter (MYB1) or a GFP reporter (MYB25 and MYB109) fused C-terminally to the respective MYB protein. The constructs were expressed under the control of either a *UBQ10* promoter (MYB1) or a *35S* promoter (MYB25 and MYB109) and tested in transgenic Arabidopsis plants (Figures 5A–C). While MYB1:YFP was located in the cytosol and the nucleus (Figure 5A), the GFP-tagged MYB25 and MYB109 proteins predominantly accumulated in the nucleus where nuclear speckles were consistently observed, suggesting that the fusion proteins are localized at sites of active transcription (Spector and Lamond, 2011).

### Overexpression of MYB25:GFP Affects Stress Tolerance and Causes Mild Developmental Changes

Based on sequence similarity and expression patterns, it seems likely that the S23 MYB family members are functionally redundant. To further evaluate *in planta* activity, we chose to characterize in greater detail (phenotypically and by RNA-seq) 35S:MYB25:GFP expressing plants. We worked with two independent transgenic lines from MYB25:GFP expressing plants, for which expression was confirmed through confocal microscopy and RT-qPCR analysis (Figure 5B and Supplementary Figure 2).

**FIGURE 6:**
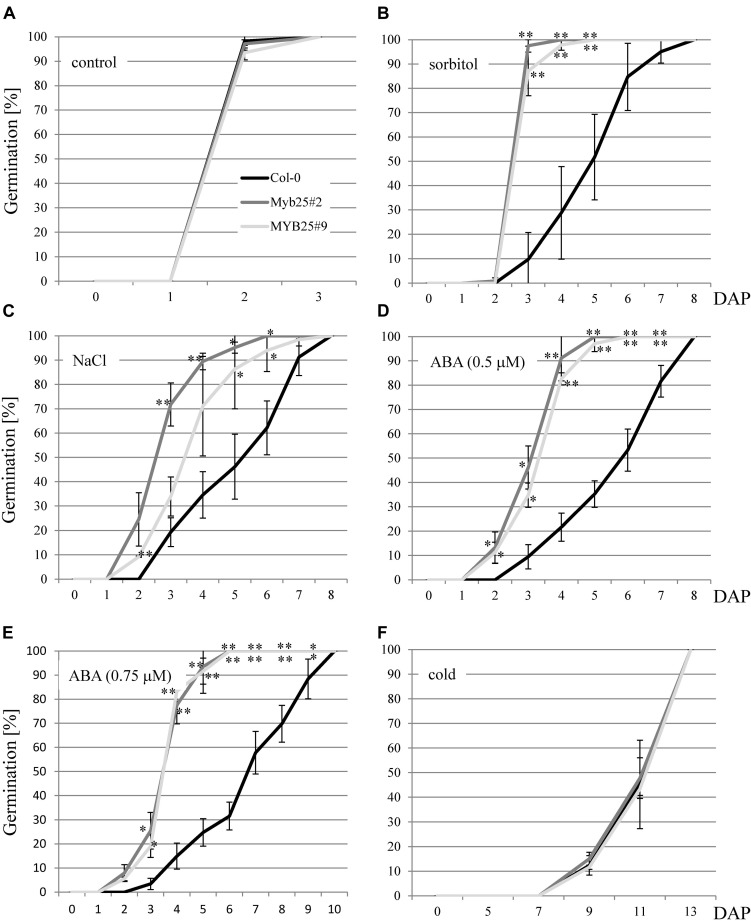
Germination of MYB25:GFP seeds in the presence of abiotic stress and ABA. **(A)** Seeds of Col-0 and two independent transgenic MYB25:GFP plants (MYB25 #2 and #9) germinate at the same rate in the absence of stress. In the presence of either 300 mM sorbitol **(B)**, 150 mM NaCl **(C)**, or ABA **(D,E)** MYB25:GFP seeds germinated significantly faster than Col-0 wild type seeds. **(F)** No difference between transgenic and Col-0 seeds were observed when incubated in the cold (4°C). In all cases at least *n* = 30 seeds were analyzed per biological replicate. DAP, days after plating. In this and subsequent figures: ^∗^, *p* < 0.05; ^∗∗^, *p* < 0.01 (student’s *t*-test). Error bars show standard deviation.

Since BPMs and other MYB transcription factors have been previously brought into context with abiotic stress responses (Jung et al., 2008; Lechner et al., 2011; Cui et al., 2013; Kim et al., 2013; Wang et al., 2015; Julian et al., 2019; Chico et al., 2020; Skiljaica et al., 2020), we analyzed MYB25:GFP overexpressor lines under abiotic stress conditions. We focused on the sensitivity of the plants at the germination stage since this is a straightforward and frequently used stage to investigate aberrant stress sensitivities in Arabidopsis. In particular, we tested osmotic (300 mM sorbitol), and salt stress (150 mM NaCl) conditions, as well as ABA treatments (0.5 and 0.75 μM). Monitoring the germination rate over time showed that MYB25:GFP seeds consistently germinated faster compared to wild type seeds on sorbitol, NaCl, and ABA (Figures 6A–E). Because ABA is connected with cold stress responses (Xiong et al., 2001; Zhu et al., 2005; Chen et al., 2009; Lu et al., 2019), we also tested our transgenic plants for germination sensitivity at 4°C, but did not observe any significant differences compared to wild type (Figure 6F).

Additionally, we generated a polycistronic artificial *amiRNA* construct that downregulates all three S23 MYBs (Supplementary Figure 3). Transgenic plants constitutively expressing the construct showed hypersensitivity toward salt and sorbitol as well as ABA at the germination stage (Supplementary Figure 4). These findings are genetically in agreement with the observed higher tolerance of the MYB25:GFP overexpression lines.

**FIGURE 7:**
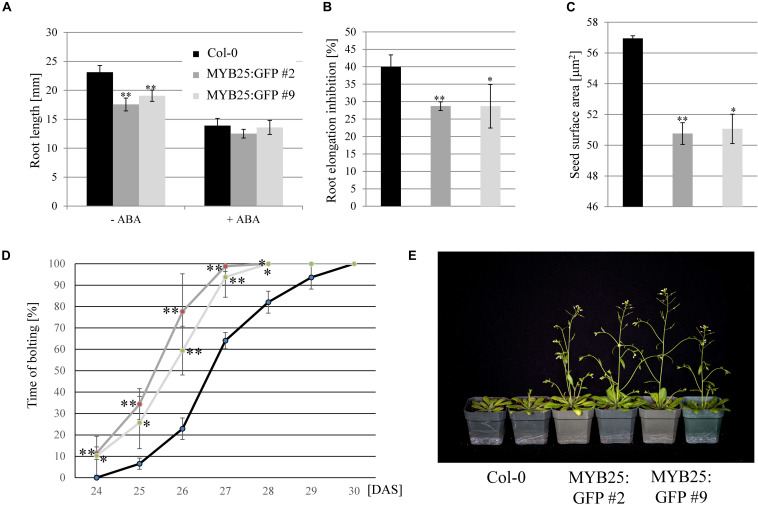
Overexpression of MYB25 affects root and seed development as well as flowering time. **(A)** MYB25:GFP plants have shorter roots compared to wild type (*n* = 30). **(B)** Presence of ABA (20 μM) causes a significantly higher reduction in root elongation in wild type plants than in the MYB25:GFP plants. **(C)** Plants with elevated *MYB25:GFP* expression develop smaller seeds compared to wild type (*n* = 30). **(D,E)** MYB25:GFP overexpressing plants show an early flowering phenotype (*n* = 20). DAS, Days After Sowing.

Sensitivities of MYB25:GFP overexpressor lines in root elongation assays was tested on salt (100 and 150 mM NaCl) and ABA (20 μM), but we only observed a significant difference to wild type on ABA (Figures 7A,B). While ABA was less effective in inhibiting elongation growth, it needs to be emphasized that MYB25:GFP seedlings already had significantly shorter roots than the wild type (Figure 7A). In addition to shorter roots, we also observed that MYB25:GFP overexpressor lines had slightly smaller seeds and an early flowering phenotype compared to wild type plants (Figures 7C–E), but were otherwise indistinguishable from wild type.

Overall, these findings indicated that MYB25 regulates osmotic and salt stress as well as ABA responses, and impacts Arabidopsis development in specific aspects, such as flowering time, seed development, and primary root length.

### MYB25 Is Involved in Regulating Stress Responses and Developmental Pathways

Because it is currently unclear what genes and processes are regulated by MYB25 in Arabidopsis, we decided to perform a transcriptional profiling on the *35S:MYB25:GFP* overexpressing seedlings. We looked separately at root and shoot tissues due to the observed differences in GUS staining (Figure 4C).

**FIGURE 8:**
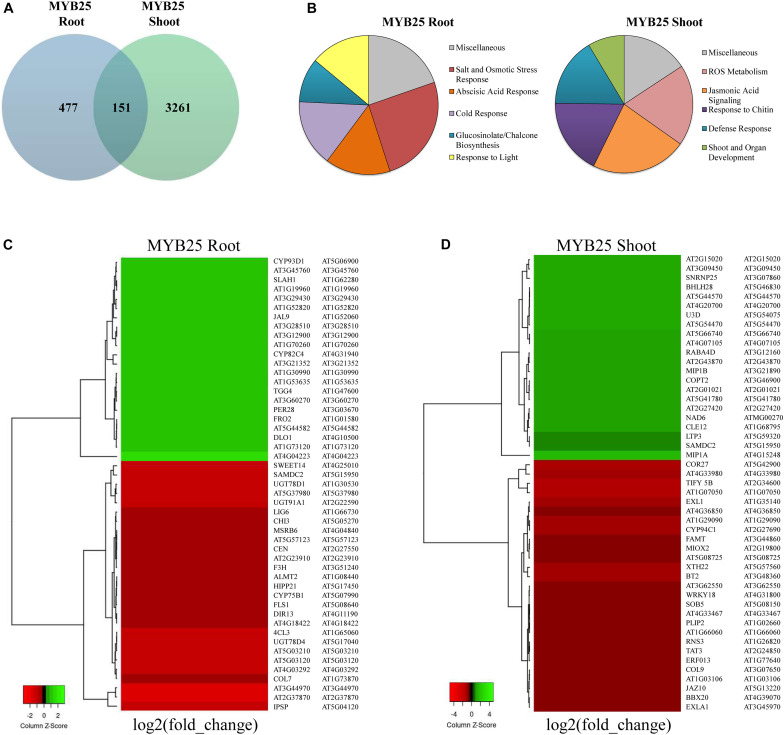
Transcriptomic profiling in roots and shoots of MYB25:GFP overexpressing seedlings. **(A)** Venn diagram showing general gene expression changes compared to Col-0 wild type. **(B)** Pie diagrams showing the most prominent changes in cellular pathways based on GO term analysis (all depicted changes represent *p* < 0.01, *q* < 0.05 for MYB25 root and shoot). **(C,D)** Heatmap of the most differentially expressed genes in root and shoot of MYB25:GFP overexpressing seedlings generated with the online tool Heatmapper (*p* < 0.01, *q* < 0.05; http://www.heatmapper.ca/).

As shown in the Venn diagram in Figure 8A, expression of a broad range of genes was significantly changed (*p* < 0.001, *q* < 0.05) in 35S:MYB25:GFP plants when compared to Col-0 wild type (for a complete list of genes see Supplementary Table 1). Interestingly, only a set of 151 genes was overlapping between shoot and root data, whereas the majority of expression changes were specific for either root or shoot. A significantly higher number of genes were changed in the shoots of 35S:MYB25:GFP plants (3261 genes), while in 35S:MYB25:GFP roots nearly seven-fold less genes (477 genes) showed aberrant expression compared to Col-0. We also verified RNA-seq data by RT-qPCR on four selected genes in independently grown 14-day old whole seedlings, and confirmed changes in gene expression (Supplementary Figure 5 and Supplementary Table 1) (log2 fold-change by RNA-seq: AT5G46830, +3.8 in shoots; AT2G30770, +2.5 in shoots; AT5G04120, −3.3 in roots; AT3G21352, 2.9 in roots). These genes were selected as they were among the most differentially regulated genes identified and demonstrate the role of MYB25 in influencing biotic and abiotic stress.

The Gene Ontology (GO) term enrichment analysis showed that a majority of the changes observed in the MYB25:GFP seedlings were related to cellular stress-response pathways, either to abiotic stress (e.g., salt, drought, osmotic, or reactive oxygen stress) or to biotic stress (e.g., jasmonic acid signaling, glucosinolate metabolism, and pathogen responses) (Figure 8B and Supplementary Table 2). For example, some of the stress responsive genes with aberrant expression in MYB25:GFP plants include slow type anion channel-associated homologue 1 (SLAH1) (Qiu et al., 2016), the dehydration-responsive element-binding protein 2C (DREB2C) (Je et al., 2014; Song et al., 2014), and responsive to dessication29a (RD29a) (Yamaguchi-Shinozaki and Shinozaki, 1994) (Supplementary Table 2). However, GO term enrichment analysis also identified changes in other processes related to, for example, general development or light response, and developmental pathways. Gene examples here include late elongated hypocotyl (LHY) (Park et al., 2016), somatic embryogenesis receptor-like kinase 4 (SERK4) (Albrecht et al., 2008), related to ABI3/VP1 1 (RAV1) (Fu et al., 2014), or circadian clock associated 1 (CCA1) (Lau et al., 2011; Park et al., 2016). GO enrichment analysis of biological pathways revealed that the roots of MYB25:GFP seedlings are altered in glucosinolate and glycosyl compound biosynthesis, while the shoots displayed aberrant biotic stress responses (Supplementary Figure 6). This is reflected among the most differentially regulated genes, as, for example, UDP-Glycosyltransferase superfamily protein members, UGT78D4 and UGT91A1 are strongly downregulated in the root, while jasmonate-zim-domain protein 10 (JAZ10) and tyrosine aminotransferase 3 (TAT3) are highly downregulated in the shoots (Figures 8C,D).

Overall, the data indicate that MYB25 fulfills distinct functions in root and shoot, and that it affects a broader range of processes in the shoot than in the root. They also show that many of the processes affected are related to stress response, while general developmental pathways are proportionally less affected, which corroborates with our phenotype observations.

## Discussion

In this study, we provide novel information about the MYB R2R3 S23 subfamily members MYB1, MYB25, and MYB109, with a closer focus on MYB25. We demonstrate for all three members tissue specific expression patterns and subcellular localization, that all three S23 members assemble with the Arabidopsis BPM family, and that they can be degraded by the 26S proteasome in cell-free degradation assays. Further we show that overexpression of *MYB25* affects an extensive range of abiotic and biotic stress response pathways at the transcriptional level and influences salt and osmotic stress, as well as ABA sensitivities. These findings are additionally supported by increased stress and ABA sensitivities in S23 knockdown mutants at the germination state caused by the polycistronic amiRNA expression. Constitutive expression of *MYB25* also impacts development, as *35S:MYB25:GFP* plants exhibit shorter roots, smaller seeds, and flower earlier.

Currently, we do not show that degradation of MYB1, MYB25, or MYB109 is facilitated by a CRL3^BPM^ E3 ligase *in planta*. However, the robust interaction with the BPMs, the well-established cell-free degradation assays we applied (Liu et al., 2017; Zhai et al., 2017; Zhao et al., 2017; Derrien et al., 2018; Selote et al., 2018), along with the *in planta* stabilization after bortezomib treatment of the different MYB:GUS proteins, suggests that this E3 ligase is facilitating ubiquitylation of all three S23 family members. We therefore consider it likely that MYB1, MYB25, and MYB109 are all targets of a CRL3^BPM^ E3 ligase and the ubiquitin proteasome pathway *in planta*. While this work extends our knowledge on the breadth of substrates targeted by this specific E3 ligase, the exact protein region that facilities the interaction between CRL3^BPM^ and its substrates remains to be determined. Direct involvement in BPM-substrate assembly has been shown for the SBC or SBC-like motifs identified in human and plants, respectively (Zhuang et al., 2009; Morimoto et al., 2017). The core motif comprises just five amino acids, often enriched in serine or threonine residues (Zhuang et al., 2009). Morimoto and co-workers showed that the motif is present in many, if not all, of the BPM substrates described in plants (Morimoto et al., 2017). Since SBC/SBC-like motifs are present in all S23 family members (Figure 1), one would expect that these are possible sites for assembly with a BPM protein. Recent work on ERF/AP2 proteins showed that a PEST motif is more critical for their interaction with BPMs and for their overall stability, rather than an SBC or SBC-like motif (Mooney et al., 2019). Since MYB1 lacks a clear PEST motif, but shows interaction and instability comparable to MYB25 and MYB109, further clarification is needed to understand on what the substrate-CRL3^BPM^ E3 ligase interaction and interplay depends.

The GUS data showed that MYB1, MYB25, and MYB109 are expressed in most tissues but often not across e.g., the complete root expression is rather restricted to specific parts such the root tip for example. This may indicate that the proteins have specific functions in root elongation or cell division for example. The instabilities of MYB1, MYB25, and MYB109 in cell-free degradation assays is generally supported by the finding that GUS protein accumulates in bortezomib treated plants, and specifically was not detectable at all in *proMYB25:MYB25:GUS* and *proMYB109:MYB109:GUS* plants without such treatment. In addition, staining needed to be done overnight to clearly see GUS activity.

We observed three clear developmental changes in the MYB25:GFP overexpression lines, which were (i) reduced root length, (ii) smaller seed size, and (iii) an early flowering phenotype. However, it should be noted that we did not see GUS activity in seeds themselves, so the aberrant development of seeds might be related to the constitutive expression of MYB25, and possibly represents an artifact due to the putative absence of MYB25 protein in seeds. In contrast, MYB25 is expressed in roots, and the shorter root in MYB25:GFP seedlings could be related to changes in meristematic activity due to the distinct GUS expression in proMYB25:MYB25:GUS plants at the root tip. Earlier reports on another CRL3^BPM^ substrate, MYB56, showed that overexpression of the transcription factor, or reduced CRL3^BPM^ activity, also results in shorter roots (Chen et al., 2013; Vilarrasa-Blasi et al., 2014). MYB56 has been described as a negative regulator of root apical meristem activity (Vilarrasa-Blasi et al., 2014), which may also be the case with MYB25. The third aberrant phenotype of MYB25:GFP plants, early flowering time, is intriguing since it characterizes MYB25 as a positive regulator of flowering time. Plants with reduced BPM levels are late flowering (Chen et al., 2013), indicating that CRL3^BPM^ E3 ligases also act as positive regulators of flowering. In contrast, MYB56 appears to be a negative regulator of this process, since the corresponding null mutants flower earlier than wild type plants (Chen et al., 2015). It will be interesting to understand how the stability of E3 ligase substrates impacts flowering time on the molecular level.

The generated gene expression profiling argues that MYB25 is involved in abiotic, as well as biotic, stress responses. Some of the genes listed, such as SLAH1, DREB2c or RD29a, are specifically connected with abiotic stress response (Yamaguchi-Shinozaki and Shinozaki, 1994; Lee et al., 2010; Bihmidine et al., 2013; Je et al., 2014; Song et al., 2014; Qiu et al., 2016). However, their expression changes do not necessarily correlate with the observed increased tolerances at the germination stage of MYB25:GFP seeds. For example RD29a is a positive mediator of drought and salt stress, but it is down-regulated in MYB25:GFP seedlings. A reason for this could be that the transcriptional changes observed in the RNA-seq approach are specific for this developmental stage, and therefore not reflecting the impact of MYB25:GFP in germinating seeds. We also did not see clear changes in stress tolerance at the seedling stage. This might be related to the overall weak expression of MYB25:GFP, which is reflected in comparably mild changes in the RNA-seq data sets. Another factor to consider is the opposing regulation of some of the stress-related genes in an organ-dependent manner (e.g., DREB2c is up-regulated in root, but down-regulated in shoot), or that some response mediators are up-while others are down-regulated in the MYB25:GFP plants (e.g., SLAH1 is up-regulated, while RD29a is down-regulated) (Supplementary Table 1). Overall, the RNA-seq data still support a role of MYB25 in stress response regulation, although the constitutive overexpression does not allow us to pinpoint which processes are directly regulated by the transcription factor, which are secondary responses, and which are more artificial due to unusual expression in certain organs, such as root, where the protein levels of the transcription factor would normally be low or non-existing.

Expression of several genes involved in cellular processes other than stress was also significantly changed in the MYB25:GFP seedlings. Notable examples here are CCA1 and LHY, which were down-regulated in the roots but up-regulated in the shoots of MYB25:GFP plants (Supplementary Table 2). LHY functions as a negative regulator of flowering, while CCA1 has been found to bind to the promoter of the floral integrator flowering locus T (FT), and to play a positive role in pathogen resistance (Park et al., 2016). CCA1/LHY double mutants have also been connected with salt, osmotic, and heat stress hypersensitivities (Kant et al., 2008).

Finally, the observed increased tolerances toward NaCl, sorbitol, and ABA at the germination stage are similar to previous works from other groups where MYB transcription factors were overexpressed. For example, Arabidopsis MYB2, a member of the S20 R2R3 subfamily (Dubos et al., 2010), enhances salt stress tolerance by interacting with calmodulin (Yoo et al., 2005), and overexpression of FtMYB13 from Tartary buckwheat improves salt and drought stress tolerance in Arabidopsis (Huang et al., 2018). Other examples within the R2R3 MYB family, such as MYB15 or MYB20, have demonstrated that these factors can also act as positive regulators of ABA signaling and abiotic stress tolerance (Ding et al., 2009; Cui et al., 2013).

In summary, our results identify the R2R3 S23 MYB transcription factors as novel regulators of biotic and abiotic stress responses that are unstable proteins targeted by the ubiquitin proteasome pathway. Based on interaction studies with BPM substrate adaptors, these findings also implicate CRL^BPM^ E3 ligases as likely to affect MYB25-dependent biotic and abiotic stress responses. Since reduced ABA, sorbitol, and salt tolerances at the germination stage resemble what was previously described for other MYB transcription factors, this may either indicate functional redundancies among these transcription factors or a coordinated effort to facilitate abiotic stress responses. More work is needed to elucidate the exact role of MYB25 in abiotic stress response and development, and to what degree the S23 family as a whole is functionally redundant in Arabidopsis.

This work further shows the role of CRL3^BPM^ activity in the regulation of plant stress response and underscores the relevance of understanding substrate interaction as a possible point in this complex, but highly conserved, pathway where modifications can be directed to improve resistance and increase yield in agriculture.

## Materials and Methods

### Plant Growth, Transformation, Stress Treatments, and Phenotyping

As plant material, *Arabidopsis thaliana* (variety Col-0) were used. Plants were grown at 20°C, with a day:night cycle of 16 h light to 8 h dark. Arabidopsis plants cultured under sterile conditions grew on minimal medium without supplemental sucrose according to Estelle and Somerville (1987). Stable Arabidopsis transformations were accomplished following the floral-dip method (Clough and Bent, 1998), while transient expression in tobacco leaves was done as described by Sparkes et al. (2006). For all phenotypical and stress related experiments, stable transgenic plants of the T3 generation were used. Salt (NaCl) and abscisic acid (ABA) (Sigma-Aldrich, St. Louis, MO, United States) dependent germination assays were performed by plating seeds on minimal medium supplemented with different NaCl or ABA concentrations, respectively. Germination was defined when radicles first emerged from the seed coat. For salt-dependent root length assays, 3-day old Arabidopsis seedlings were transferred individually to plates supplemented with or without different NaCl concentrations. Growth of the primary root was measured daily for up to 2 weeks. For gene expression analysis in conjunction with salt stress, we adopted a protocol described in Wang et al. (2015). In brief, 9-day-old Col-0 seedlings, grown on vertical minimal media plates containing 1% agarose, were transferred to 5 mL minimal media solution supplemented with or without 200 mM NaCl for 4 h. Quantification of seed size was done using ImageJ software (Schneider et al., 2012). For flowering time points, seeds were directly brought out into soil, and transferred to individual pots 10 days later. Beginning of flowering was defined when first inflorescences became visible (time of bolting). All experiments were repeated at least three times independently as biological replicates.

### Generation of Constructs

Any genetic material for MYB1, MYB25, and MYB109 used in this work was amplified either from Col-0 genomic DNA or cDNA generated from total RNA via a High Capacity cDNA Reverse Transcription kit according to the kit’s manual (Applied Biosystems, Foster City, CA, United States). PCR-based amplification of DNA was accomplished with Phusion High-Fidelity DNA Polymerase under standard conditions (Thermo Fisher Scientific, Waltham, MA, United States). For the MYB1 and *MYB25* promoters a 1600-bp long region upstream of the ATG was amplified, while for the *MYB109* promoter this comprised 1592 bp. PCR products were first sub-cloned into *pCR8* (LifeScience, Darmstadt, Germany) and sequenced before being further processed. For expression of GST-fusion proteins in *Escherichia coli*, *MYB1*, *MYB25*, *MYB56*, and *MYB109* cDNAs were shuffled via LR-GATEWAY-reactions to *pDEST15* (LifeScience, Darmstadt, Germany). A cDNA construct received from the Arabidopsis Stock Center Resource for MYB1 (DKLAT3G09230) was cloned into *pHB2-GST* (ABRC, Columbus, OH, United States) using *Nde*I/*Eco*RI sites. For expression of His-tagged BPM1, 3, 5 proteins, the corresponding gene was cloned into *pET21b* (Novagen Inc., Madison, WI, United States) using the *Nde*I/*Xho*I restriction sites. For *in planta* expression of C-terminally tagged MYB1:YFP fusion proteins under the control of a *UBQ10* promoter was cloned into the *Xma*I/*BamH*I sites of the modified *pGREEN* vector *pG20-YFP* (Pratt et al., 2020). C-terminally tagged MYB25: and MYB109:GFP fusion proteins under the control of a 35S promoter, and C-terminal GFP constructs using the genomic DNA regions (introns and exons but no promoter) of MYB25 and MYB109 were generated in the Gateway-compatible vectors *pMDC43* and *pMDC83*, respectively (Curtis and Grossniklaus, 2003). For GUS reporter assays, a promoter genomic *MYB1* DNA region (introns and exons plus promoter) was cloned in the modified *pGREEN* vector *pG20-GUS* (Pratt et al., 2020) via Gibson technology using the *Hind*III/*Xma*I sites. Promoter:genomic constructs for *MYB25* and *MYB109* were cloned via Gateway technology using the destination vector *pMDC163* (Curtis and Grossniklaus, 2003). A polycistronic artificial microRNA (amiRNA) construct with two independent amiRNAs was generated based on predictions from the WMD3 server^5^. One amiRNA targets *MYB25* and *MYB109* (CAGCGCTCTGGTTAATCTTT), while the second one targets MYB1 and MYB25 (TAAGATTTACCAGAGCGCCGT). No off targets were predicted. The two amiRNAs are co-expressed, and separated by RNASe P and Z cutting site according to Xie et al. (2015), that allow *in planta* processing to gain individual amiRNAs. The two amiRNAs are followed by an active ribosomal complimentary sequence (ARC) which functions as internal ribosome entry site to allow uncapped translation of any protein behind the ARC sequence (Chappell et al., 2000; Akbergenov et al., 2004; Pfeiffer et al., 2012). The construct was synthesized by Bio Basic Inc., Amherst, NY, United States, and cloned into the modified *pGREEN* vector *gG20-GUS* (Pratt et al., 2020) via Gibson technology using the *Sma*I/*Bam*HI. The vector allows constitutive expression based on a *UBQ10* promoter, and easy tracking of expression using its GUS reporter (Supplementary Figure 3). All primers used in this work are listed in Supplementary Table 3.

### Expression and Purification of Recombinant Proteins

Recombinant proteins were expressed and purified using standard methods as described earlier (Chen et al., 2013). In brief, protein cultures were grown overnight at 37°C, diluted to an OD_600_ of 0.1–0.3, and grown to an OD_600_ of 0.6–0.8. The cultures were then induced with 0.1 M IPTG (Isopropyl β-D-1-thiogalactopyranoside), grown for an additional 2 h, pelleted, and stored at −20°C before protein extraction. Expressed proteins were affinity purified from *E. coli* via glutathione agarose beads (Sigma-Aldrich, St. Louis, MO, United States), or via Ni-NTA agarose beads (Sigma-Aldrich, St. Louis, MO, United States). For elution of GST-tagged recombinant proteins from beads, proteins were incubated for 1h at 4°C in GST-elution buffer (25 mM reduced glutathione; 50 mM Tris pH 8.8; 200 mM NaCl).

### Gus Staining and Expression Analysis

Tissue specific expression patterns of GUS reporter constructs introduced to Arabidopsis were assessed according to Jefferson (1989). In brief, plant tissues were harvested at various ages and incubated if not otherwise stated in GUS staining solution for 12 h at 37°C. Tissue was cleared using 70% ethanol and incubated in 50% ethanol prior to microscopic imaging. Plant tissues were imaged using a Leitz fluorescence stereomicroscope and a Leica MZ8 Camera. For detection of GUS protein, an α-β-Glucuronidase (N-Terminal) antibody (Sigma-Aldrich, St. Louis, MO, United States) was used. For bortezomib (ApexBio, Houston, TX, United States) treatment plants were incubated in liquid minimal medium supplemented with the inhibitor as indicated in the figure and legend for the duration of the experiment.

### Subcellular Localization Studies via Confocal Microscopy

The constructs were initially screened for localization using transient expression in *Nicotiana benthamiana* epidermal cells as a preliminary assay as described by Chen et al. (2015). Subsequently, transgenic *A. thaliana* seedling roots were screened for GFP expression. Arabidopsis tissues were imaged using a Leica SP8 confocal compound microscope.

### Pulldown Assays

N-terminal GST-tagged MYB proteins were purified and eluted before incubated with His:BPM1, :BPM3, and :BPM5 that remained on Ni-NTA agarose beads in a Tris Buffer (0.1 M Tris pH 7.5, 150 mM NaCl, 0.5% NP-40 (Igepal CA-630) containing 100 mM PMSF protease inhibitor for 2 h at 4°C followed by three ten minute washes. Samples were boiled in a 4× SDS loading buffer (0.25 M Tris pH 6.8, 8% SDS, 20% β-mercaptoethanol), run on a 10% SDS-PAGE gel, and transferred to a 0.45 μm PVDF membrane (TISCH Scientific, North Bend, OH, United States) for immunodetection via monoclonal GST and His antibodies (LifeTein, Somerset, NJ, United States), and a horse radish-coupled secondary donkey anti-mouse (Santa Cruz Biotechnology, Dallas, TX, United States). Samples were initially detected using an α-GST antibody and stripped and re-probed using an α-His antibody.

### Yeast 2-Hybrid Assays

Yeast 2-Hybrid studies were done as described earlier (Weber et al., 2005). Selection medium II (SDII) was supplemented with histidine and uracil and represented a transformation control. SDIV minimal medium lacked uracil and histidine and was used for interaction studies. Photos were taken from individual spots 1 week after plating.

### Cell-Free Degradation Assays

Cell-free degradation assays generally followed a protocol described by Wang et al. (2009). In brief, a master mix that allowed 20 ul samples to be removed at each time point of eluted recombinant GST-tagged proteins (100 ng) were incubated in native plant extract (200 μg) from 12-day-old *A. thaliana* seedlings (Chen et al., 2013). GST-tagged proteins were incubated in a plant buffer (4 mM PMSF, 10 mM ATP, and 5 mM DTT) rocking at room temperature, with samples taken at 0, 30, and 60 min. An additional sample was treated with the proteasome inhibitor MG132 (20 μM; Selleckchem, Houston, TX, United States), and incubated for the duration of the experiment.

### RNA Extraction, RT-PCR, and RT-qPCR

Approximately 50 mg of tissue was frozen in liquid nitrogen and ground using a mortar and pestle. RNA was extracted from *Arabidopsis* tissues using a RNeasy Plant Mini RNA Kit (Qiagen, Germantown, MD, United States) with an RLT lysis buffer as described by the manufacturer. Reverse transcription was performed using an Applied Biosystems high-capacity kit for reverse transcription into cDNA. The cDNA was either used for basic PCR (GFP, DDB1a) or for RT-qPCR (*MYB1*, *MYB25*, *MYB109*, *ACTIN2*, *BHLH28*, *CYP71A13*, *dPGM*, *AT3G21352*). For expression analysis by RT-qPCR an ABI 7500 Fast Real Time PCR machine was used. All primers used are listed in Supplementary Table 3.

### RNA-Seq Experiments and Data Analysis

Overexpression lines for MYB25:GFP, along with Col-0, were grown vertically in sterile culture on minimal medium and harvested at 14-days after plating. Seedling roots and shoots were separated for each line and used for RNA extraction with a RNeasy Plant Mini RNA (Qiagen, Germantown, MD, United States) extraction kit. RNA extractions were collected in triplicates. RNA sequencing and quality control were done at the Oregon State University’s Center for Genome Research and Biocomputing with each sample barcoded, pooled, and split between two Illumina HiSeq 3000 lanes for sequencing (150 bp single end). The barcodes from each sample were trimmed, and the samples were filtered for quality. Sequencing data were processed using the Tuxedo Suite and analyzed using the protocol from Trapnell et al. (2012). In brief, reads were aligned with Top Hat against the TAIR10 *A. thaliana* genome, and transcripts were assembled using Cufflinks. The reads were analyzed for differential gene expression using Cuffdiff. Visualization of data was performed using Cummerbund on significant results (*p* < 0.01, *q* < 0.05 for MYB25 root and shoot). GO analysis was performed using GOTermFinder^6^. The most differentially regulated GO processes were selected for the creation of pie charts and heatmaps. Further heatmaps were generated with HeatMapper^7^ expression plots. Additional GO enrichment analysis of the top 30 differentially regulated biological process pathways was performed using ShinyGO v0.66^8^ to demonstrate the interplay of these GO categories (*p* < 0.05).

## Data Availability Statement

The RNA-seq datasets are deposited to the NCBI Sequence Read Archive and have the BioProject accession number PRJNA679829.

## Author Contributions

HH, CB, and SM designed the project. CB performed the confocal microscopy on MYB25: and MYB109:GFP, instability data, generation of MYB25 and MYB109 expressing transgenic plants, *MYB25* and *MYB109* GUS reporter analysis, seed phenotyping, as well as RNA-seq analysis, and presentation of all RNA-seq data, while HH and SM provided guidance. SM participated in construct generation, germination, and flowering time experiments, and performed all interaction studies (Y2H and pulldown), RT-qPCR, MYB1 GUS reporter analysis, and seed germination analysis. RA-S did the ABA root elongation assays. AG provided help and advice for the transcriptional profiling. HH generated the *MYB1* (*GUS* and *GFP*) and the polycistronic *amiMYB* expression constructs and the corresponding plants, wrote the manuscript, generated all figures except for the RNA-seq data, and assisted where needed. All authors edited and reviewed the manuscript for submission.

## Conflict of Interest

The authors declare that the research was conducted in the absence of any commercial or financial relationships that could be construed as a potential conflict of interest.

## Publisher’s Note

All claims expressed in this article are solely those of the authors and do not necessarily represent those of their affiliated organizations, or those of the publisher, the editors and the reviewers. Any product that may be evaluated in this article, or claim that may be made by its manufacturer, is not guaranteed or endorsed by the publisher.

## Footnotes

1
https://www.arabidopsis.org/servlets/TairObject?id=37199&type=locus

2
http://elm.eu.org/

3
http://emboss.bioinformatics.nl/cgi-bin/emboss/epestfind

4
https://bio.tools/UbPred

5
http://wmd3.weigelworld.org/cgi-bin/webapp.cgi

6
https://go.princeton.edu/cgi-bin/GOTermFinder

7
http://www.heatmapper.ca/

8
http://bioinformatics.sdstate.edu/go/
