# Supplementary figures and images for: Characterization of Arabidopsis thaliana R2R3 S23 MYB Transcription Factors as Novel Targets of the Ubiquitin Proteasome-Pathway and Regulators of Salt Stress and Abscisic Acid Response
Source: Front Plant Sci. 2021 Aug 19;12:629208. doi: 10.3389/fpls.2021.629208 (PMC8417012; doi:10.3389/fpls.2021.629208)

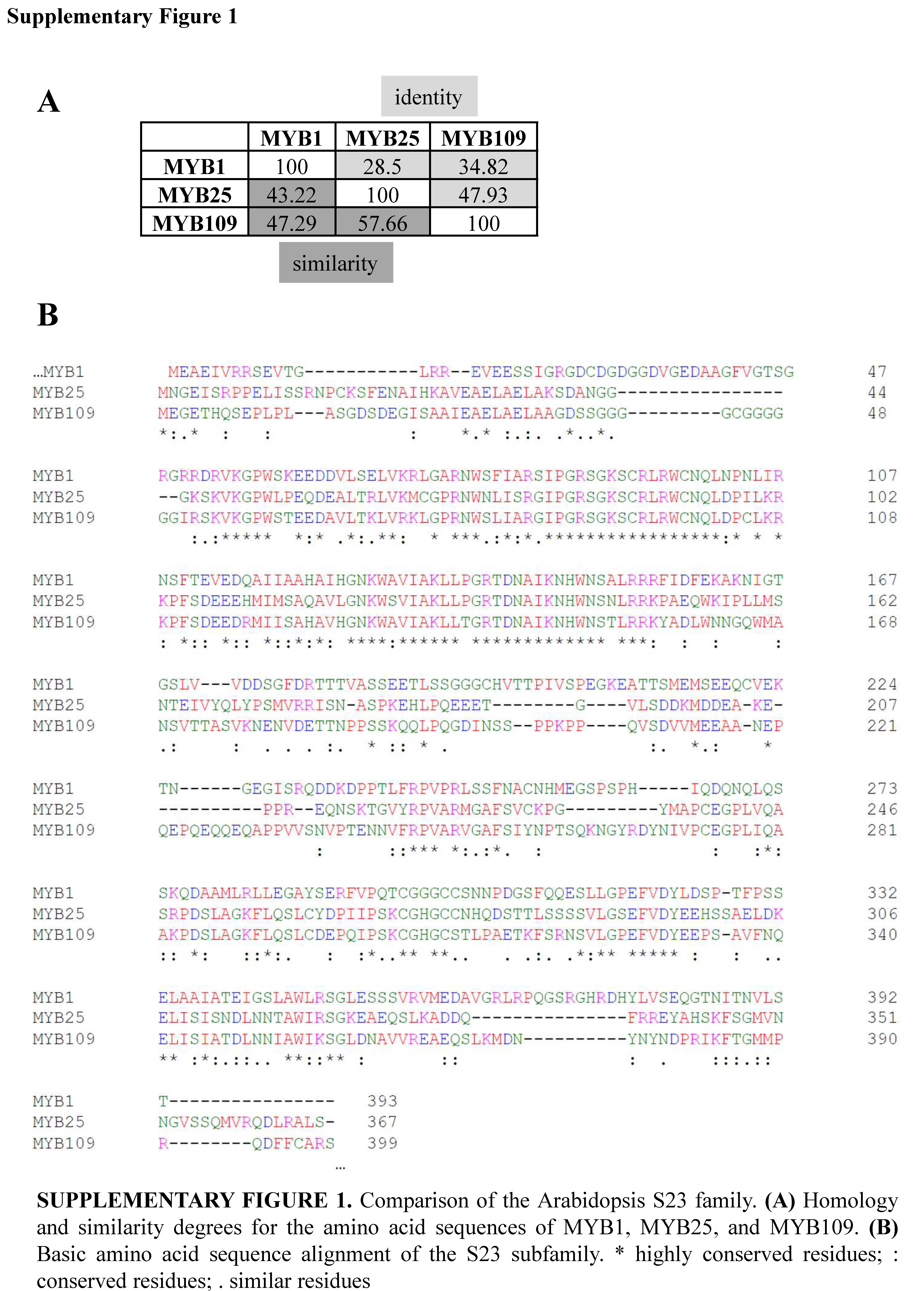

Supplement: Supplementary file 1 [file Image_1.JPEG]

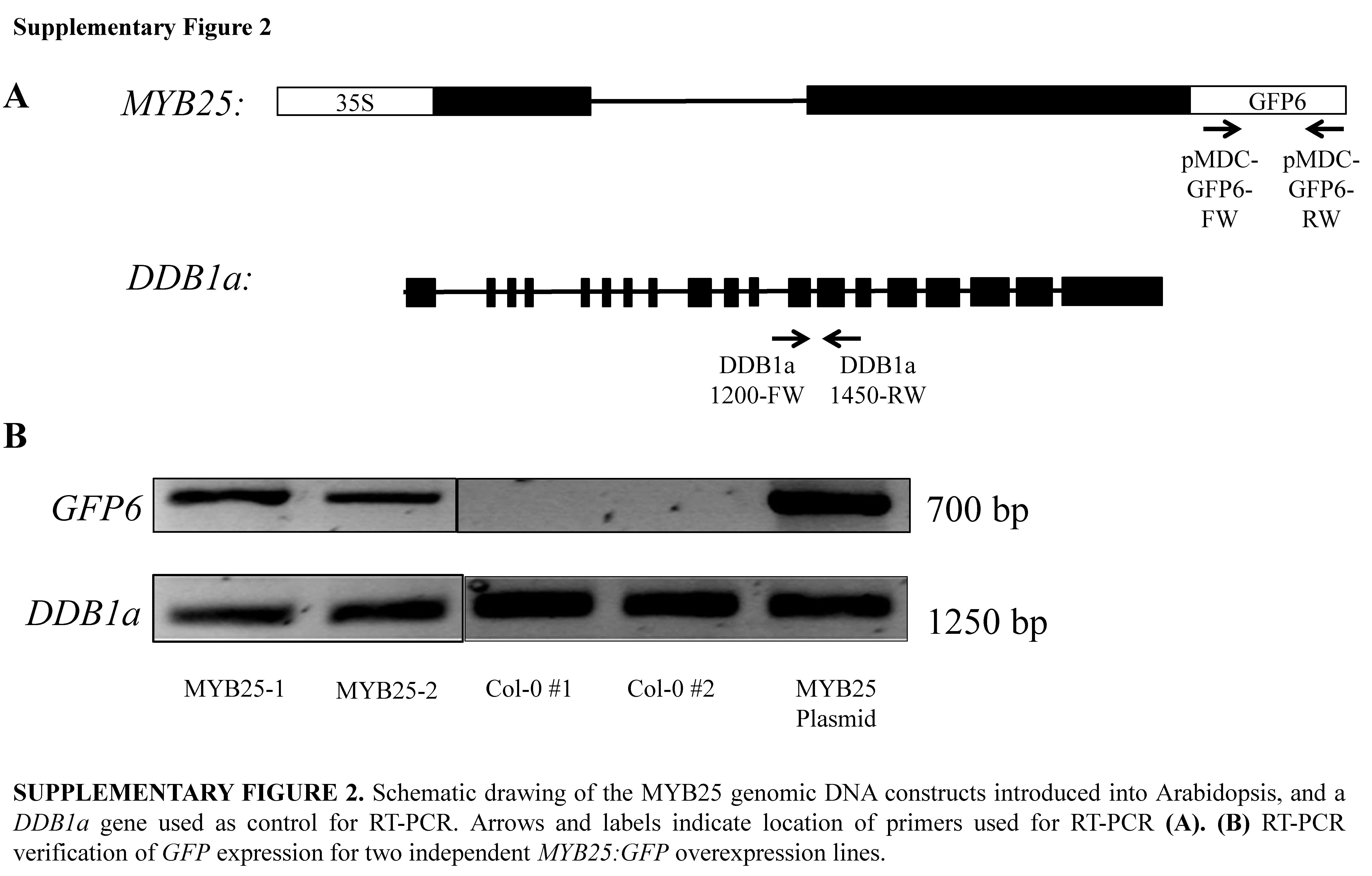

Supplement: Supplementary file 2 [file Image_2.JPEG]

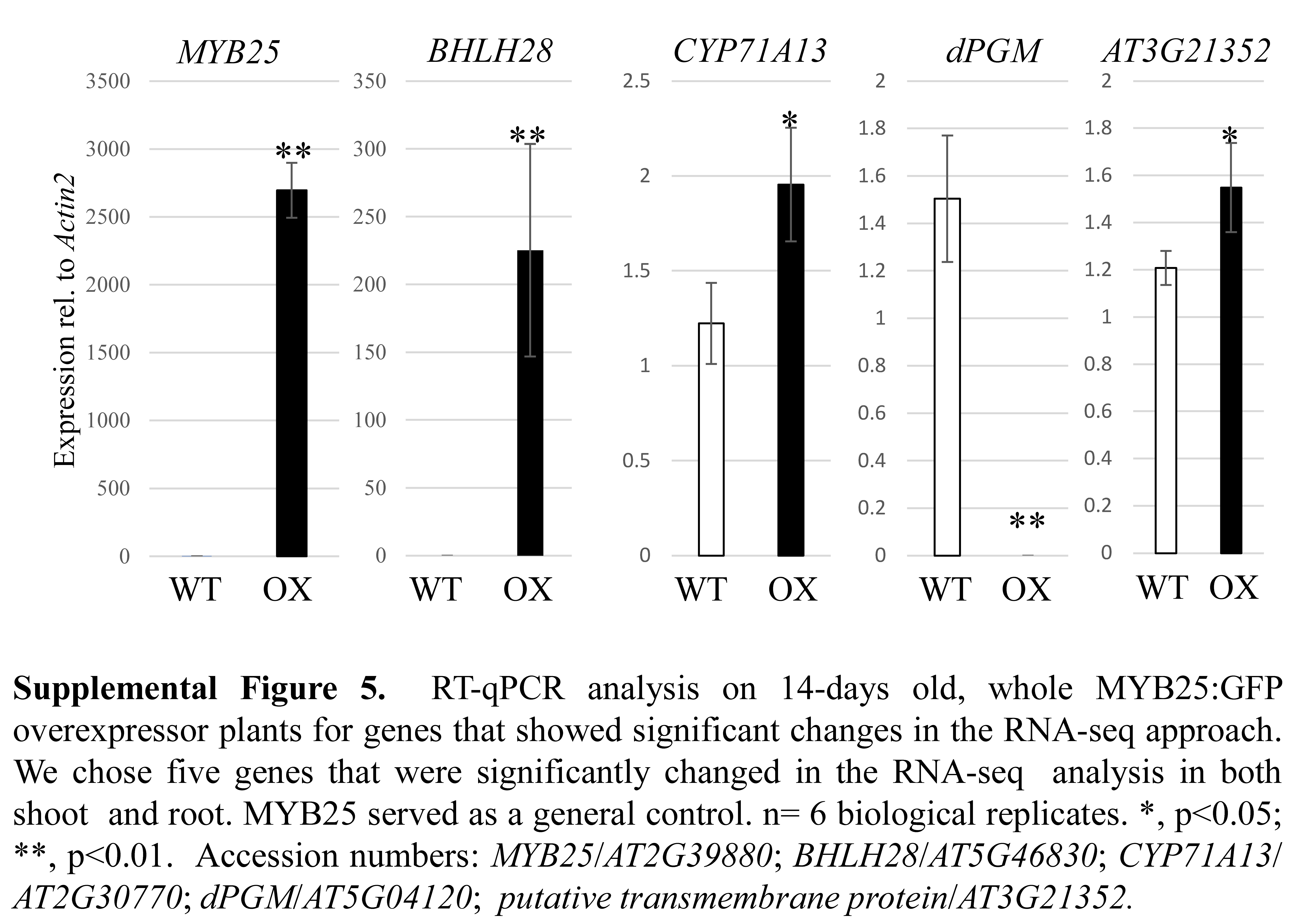

Supplement: Supplementary file 5 [file Image_5.jpg]

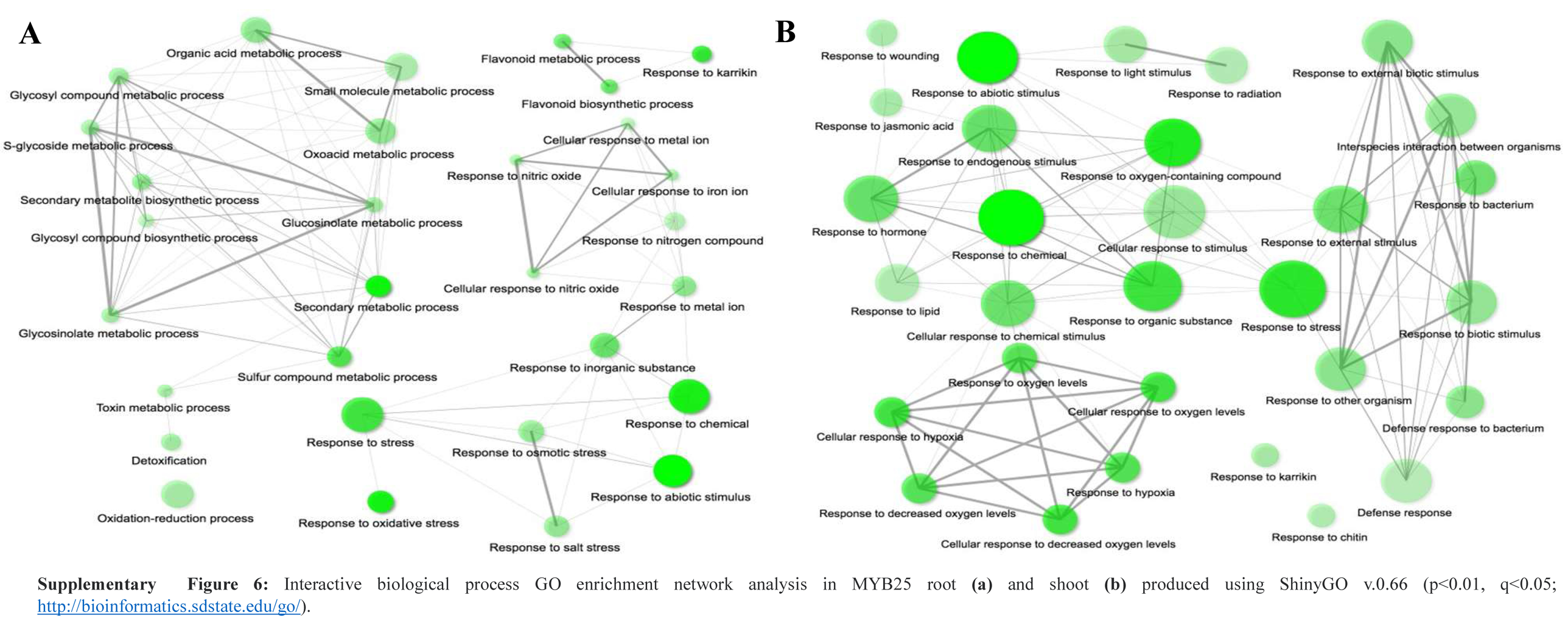

Supplement: Supplementary file 6 [file Image_6.JPEG]
