# Supplementary material for: Characterization of Arabidopsis thaliana R2R3 S23 MYB Transcription Factors as Novel Targets of the Ubiquitin Proteasome-Pathway and Regulators of Salt Stress and Abscisic Acid Response
Source: Front Plant Sci. 2021 Aug 19;12:629208. doi: 10.3389/fpls.2021.629208 (PMC8417012; doi:10.3389/fpls.2021.629208)
Supplement: Supplementary file 8 [file Table_2.DOCX]

**Supplementary Table 2:** Representative list of genes with significant changes in expression in the root and shoot of MYB25 overexpressing plants from the most mis-regulated GO processes with a focus on stress-related processes. The heat map indicates in green elevated expression, while red shows reduced expression. Note: all changes were significant with p<0.001 and q<0.05 values.

| **MYB25 Root: Salt and Osmotic Stress Response** | | | | |
| --- | --- | --- | --- | --- |
| **Gene** | **At #** | **log2(fold_change)** | **p_value** | **q_value** |
| SLAH1 | AT1G62280 | 2.49446 | 0.00015 | 0.0123286 |
| DREB2C | AT2G40340 | 1.83554 | 0.00005 | 0.00544407 |
| JUB1 | AT2G43000 | 1.77746 | 0.00005 | 0.00544407 |
| RD19A | AT4G39090 | 1.25347 | 0.00005 | 0.00544407 |
| HEL | AT3G04720 | 1.11791 | 0.00005 | 0.00544407 |
| GSTF6 | AT1G02930 | 1.06175 | 0.0004 | 0.0248506 |
| HHP1 | AT5G20270 | -0.931587 | 0.00115 | 0.0496758 |
| RD29A | AT5G52310 | -0.957992 | 0.00015 | 0.0123286 |
| UGT74E2 | AT1G05680 | -1.00315 | 0.0005 | 0.0292562 |
| ANN3 | AT2G38760 | -1.0541 | 0.0002 | 0.0149279 |
| RVE8 | AT3G09600 | -1.06598 | 0.0003 | 0.0201171 |
| SAL1 | AT5G63980 | -1.10954 | 0.00005 | 0.00544407 |
| CCA1 | AT2G46830 | -1.13219 | 0.00005 | 0.00544407 |
| ROSY1 | AT2G16005 | -1.17926 | 0.0001 | 0.00922402 |
| BGLU24 | AT5G28510 | -1.52662 | 0.00045 | 0.0270038 |
| LSU2 | AT5G24660 | -1.80806 | 0.00005 | 0.00544407 |
| LHY | AT1G01060 | -1.82227 | 0.00005 | 0.00544407 |

| **MYB25 Root: ABA Response** | | | | |
| --- | --- | --- | --- | --- |
| **Gene** | **At #** | **log2(fold_change)** | **p_value** | **q_value** |
| SLAH1 | AT1G62280 | 2.49446 | 0.00015 | 0.0123286 |
| DREB2C | AT2G40340 | 1.83554 | 0.00005 | 0.00544407 |
| AATP1 | AT1G80300 | 1.42525 | 0.00005 | 0.00544407 |
| RD19A | AT4G39090 | 1.25347 | 0.00005 | 0.00544407 |
| HEL | AT3G04720 | 1.11791 | 0.00005 | 0.00544407 |
| PCAP2 | AT5G44610 | 0.998831 | 0.0001 | 0.00922402 |
| PYL4 | AT2G38310 | 0.837993 | 0.0011 | 0.048206 |
| TTL1 | AT1G53300 | -0.829907 | 0.00085 | 0.0405295 |
| HHP1 | AT5G20270 | -0.931587 | 0.00115 | 0.0496758 |
| RD29A | AT5G52310 | -0.957992 | 0.00015 | 0.0123286 |
| UGT74E2 | AT1G05680 | -1.00315 | 0.0005 | 0.0292562 |
| TGG1 | AT5G26000 | -1.04194 | 0.0002 | 0.0149279 |
| ANN3 | AT2G38760 | -1.0541 | 0.0002 | 0.0149279 |
| RVE8 | AT3G09600 | -1.06598 | 0.0003 | 0.0201171 |
| SAL1 | AT5G63980 | -1.10954 | 0.00005 | 0.00544407 |
| CCA1 | AT2G46830 | -1.13219 | 0.00005 | 0.00544407 |
| ANNAT7 | AT5G10230 | -1.23727 | 0.00005 | 0.00544407 |
| LSU2 | AT5G24660 | -1.80806 | 0.00005 | 0.00544407 |
| LHY | AT1G01060 | -1.82227 | 0.00005 | 0.00544407 |

| **MYB25 Root: Glucosinolate/Chalcone Biosynthesis** | | | | |
| --- | --- | --- | --- | --- |
| **Gene** | **At #** | **log2(fold_change)** | **p_value** | **q_value** |
| CYP82C4 | AT4G31940 | 2.75971 | 0.00005 | 0.00544407 |
| TGG4 | AT1G47600 | 1.99374 | 0.00025 | 0.017544 |
| TGG5 | AT1G51470 | 1.7812 | 0.00055 | 0.0306534 |
| JUB1 | AT2G43000 | 1.77746 | 0.00005 | 0.00544407 |
| SOT16 | AT1G74100 | -1.36579 | 0.00005 | 0.00544407 |
| IGMT5 | AT1G76790 | -1.47786 | 0.00005 | 0.00544407 |
| BGLU24 | AT5G28510 | -1.52662 | 0.00045 | 0.0270038 |
| CHS | AT5G13930 | -1.61277 | 0.00005 | 0.00544407 |
| SAMDC2 | AT5G15950 | -2.60773 | 0.00005 | 0.00544407 |

| **MYB25 Root: Response to Light** | | | | | |
| --- | --- | --- | --- | --- | --- |
| **Gene** | **At #** | **log2(fold_change)** | **p_value** | | **q_value** |
| GRP5 | AT3G20470 | 1.79997 | 0.00005 | | 0.00544407 |
| AATP1 | AT1G80300 | 1.42525 | 0.00005 | | 0.00544407 |
| AGL14 | AT4G11880 | 1.32674 | 0.00005 | | 0.00544407 |
| ECS1 | AT1G31580 | -1.38686 | 0.00015 | | 0.0123286 |
| BGLU24 | AT5G28510 | -1.52662 | 0.00045 | | 0.0270038 |
| KIN1 | At5g15960 | -1.57873 | 0.00005 | | 0.00544407 |
| ELIP1 | AT3G22840 | -1.65475 | 0.00005 | | 0.00544407 |
| LHY | AT1G01060 | -1.82227 | 0.00005 | | 0.00544407 |
| CYP75B1 | AT5G07990 | -2.05078 | 0.00005 | | 0.00544407 |
| COL7 | AT1G73870 | -2.11691 | 0.001 | | 0.0452313 |
| 4CL3 | AT1G65060 | -2.18353 | 0.00005 | | 0.00544407 |
| UGT78D1 | AT1G30530 | -2.4189 | 0.00005 | | 0.00544407 |
| **MYB25 Root: Cold Response** | | | | | |
| **Gene** | **At #** | **log2(fold_change)** | **p_value** | **q_value** | |
| DREB2C | AT2G40340 | 1.83554 | 0.00005 | 0.00544407 | |
| GRP5 | AT3G20470 | 1.79997 | 0.00005 | 0.00544407 | |
| TTL1 | AT1G53300 | -0.829907 | 0.00085 | 0.0405295 | |
| HHP1 | AT5G20270 | -0.931587 | 0.00115 | 0.0496758 | |
| ANN3 | AT2G38760 | -1.0541 | 0.0002 | 0.0149279 | |
| SAL1 | AT5G63980 | -1.10954 | 0.00005 | 0.00544407 | |
| ANNAT7 | AT5G10230 | -1.23727 | 0.00005 | 0.00544407 | |
| KIN1 | At5g15960 | -1.57873 | 0.00005 | 0.00544407 | |
| LSU2 | AT5G24660 | -1.80806 | 0.00005 | 0.00544407 | |

| **MYB25 Root: Miscellaneous** | | | | |  |
| --- | --- | --- | --- | --- | --- |
| **Gene** | **At #** | **log2(fold_change)** | **p_value** | **q_value** | |
| JAL9 | At1g52060 | 2.59399 | 0.00005 | 0.00544407 |  |
| CYP93D1 | AT5G06900 | 2.45189 | 0.00105 | 0.0467915 |  |
| PER28 | At3g03670 | 2.08728 | 0.00005 | 0.00544407 |  |
| PER59 | At5g19890 | 1.49621 | 0.00005 | 0.00544407 |  |
| ALT3 | AT1G68260 | 1.36706 | 0.0009 | 0.0419662 |  |
| CWINV6 | AT5G11920 | 0.893839 | 0.0008 | 0.0388469 |  |
| HSP70-3 | At3g09440 | -1.47902 | 0.00005 | 0.00544407 |  |
| PIN5 | AT5G16530 | -1.5382 | 0.00005 | 0.005444 |  |
| CHI1 | At3g55120 | -1.84521 | 0.00005 | 0.00544407 |  |
| CHI3 | At5g05270 | -1.88106 | 0.00005 | 0.00544407 |  |
| MSRB6 | AT4G04840 | -1.93432 | 0.00005 | 0.00544407 |  |
| UGT78D4 | At5g17040 | -2.1816 | 0.00005 | 0.00544407 |  |
| UGT91A1 | At2g22590 | -2.47005 | 0.00005 | 0.00544407 |  |

| **MYB25 Shoot: Defense Response** | | | | |
| --- | --- | --- | --- | --- |
| **Gene** | **At #** | **log2(fold_change)** | **p_value** | **q_value** |
| CLE12 | At1g68795 | 3.0648 | 0.00295 | 0.0232388 |
| CYP71A13 | AT2G30770 | 2.52489 | 0.0027 | 0.0216738 |
| LDOX | AT4G22880 | 2.1626 | 0.00005 | 0.000829337 |
| HEL | AT3G04720 | -0.551425 | 0.0055 | 0.0374344 |
| GSTF7 | AT1G02920 | -0.838253 | 0.0001 | 0.00152628 |
| RD19A | AT4G39090 | -0.906086 | 0.00015 | 0.00214411 |
| GSTF6 | AT1G02930 | -1.24109 | 0.00005 | 0.000829337 |
| GSTU4 | AT2G29460 | -1.2851 | 0.00095 | 0.00950891 |
| TAT3 | AT2G24850 | -2.85342 | 0.00035 | 0.00435491 |
| WRKY18 | AT4G31800 | -2.90766 | 0.00005 | 0.000829337 |
| PLIP2 | AT1G02660 | -2.96077 | 0.00005 | 0.000829337 |
| JAZ10 | AT5G13220 | -3.09429 | 0.00005 | 0.000829337 |
| FAMT | AT3G44860 | -3.33914 | 0.00005 | 0.000829337 |
| XTH22 | AT5G57560 | -3.85728 | 0.00005 | 0.000829337 |
| COR27 | AT5G42900 | -4.35397 | 0.00005 | 0.000829337 |

| **MYB25 Shoot: ROS Metabolism** | | | | | | | | | |  |
| --- | --- | --- | --- | --- | --- | --- | --- | --- | --- | --- |
| **Gene** | | | **At #** | | **log2(fold_change)** | | **p_value** | **q_value** | |  |
| MIP1A | | | AT3G21890 | | 5.46083 | | 0.00115 | 0.0110262 | |  |
| BHLH28 | | | AT5G46830 | | 3.84651 | | 0.00005 | 0.000829337 | |  |
| SAMDC2 | | | AT5G15950 | | 2.98766 | | 0.00005 | 0.000829337 | |  |
| CYP71A13 | | | AT2G30770 | | 2.52489 | | 0.0027 | 0.0216738 | |  |
| TRY | | | AT5G53200 | | 2.44367 | | 0.0001 | 0.00152628 | |  |
| RGL2 | | | AT3G03450 | | 2.00591 | | 0.00005 | 0.000829337 | |  |
| MYB29 | | | AT5G07690 | | 1.86067 | | 0.00005 | 0.000829337 | |  |
| PYL6 | | | AT2G40330 | | -0.566334 | | 0.0067 | 0.0433873 | |  |
| GSTF7 | | | AT1G02920 | | -0.838253 | | 0.0001 | 0.00152628 | |  |
| RD19A | | | AT4G39090 | | -0.906086 | | 0.00015 | 0.00214411 | |  |
| DREB2C | | | AT2G40340 | | -1.03256 | | 0.00025 | 0.0033076 | |  |
| WRKY48 | | | At5g49520 | | -1.79781 | | 0.00005 | 0.000829337 | |  |
| BCB | | | AT5G20230 | | -2.75827 | | 0.00005 | 0.000829337 | |  |
| TAT3 | | | AT2G24850 | | -2.85342 | | 0.00035 | 0.00435491 | |  |
| WRKY18 | | | AT4G31800 | | -2.90766 | | 0.00005 | 0.000829337 | |  |
| PLIP2 | | | AT1G02660 | | -2.96077 | | 0.00005 | 0.000829337 | |  |
| COL9 | | | AT3G07650 | | -3.07328 | | 0.00005 | 0.000829337 | |  |
| FAMT | | | AT3G44860 | | -3.33914 | | 0.00005 | 0.000829337 | |  |
| BT2 | | | AT3G48360 | | -3.72615 | | 0.00005 | 0.000829337 | |  |
| **MYB25 Shoot: Response to Chitin** | | | | | | | | | | |
| **Gene** | | | | **At #** | **log2(fold_change)** | | **p_value** | | **q_value** | |
| MIP1A | | | | AT3G21890 | 5.46083 | | 0.00115 | | 0.0110262 | |
| BHLH28 | | | | AT5G46830 | 3.84651 | | 0.00005 | | 0.000829337 | |
| SAMDC2 | | | | AT5G15950 | 2.98766 | | 0.00005 | | 0.000829337 | |
| CYP71A13 | | | | AT2G30770 | 2.52489 | | 0.0027 | | 0.0216738 | |
| LDOX | | | | AT4G22880 | 2.1626 | | 0.00005 | | 0.000829337 | |
| MYB29 | | | | AT5G07690 | 1.86067 | | 0.00005 | | 0.000829337 | |
| GSTF7 | | | | AT1G02920 | -0.838253 | | 0.0001 | | 0.00152628 | |
| RD19A | | | | AT4G39090 | -0.906086 | | 0.00015 | | 0.00214411 | |
| GSTF6 | | | | AT1G02930 | -1.24109 | | 0.00005 | | 0.000829337 | |
| WRKY48 | | | | At5g49520 | -1.79781 | | 0.00005 | | 0.000829337 | |
| WRKY18 | | | | AT4G31800 | -2.90766 | | 0.00005 | | 0.000829337 | |
| PLIP2 | | | | AT1G02660 | -2.96077 | | 0.00005 | | 0.000829337 | |
| BBX20 | | | | AT4G39070 | -3.02987 | | 0.00005 | | 0.000829337 | |
| COL9 | | | | AT3G07650 | -3.07328 | | 0.00005 | | 0.000829337 | |
| BT2 | | | | AT3G48360 | -3.72615 | | 0.00005 | | 0.000829337 | |
| **MYB25 Shoot: ROS Catabolism** | | | | | | | | | | |
| **Gene** | | **At #** | | | **log2(fold_change)** | | **p_value** | | **q_value** | |
| MIP1A | | AT3G21890 | | | 5.46083 | | 0.00115 | | 0.0110262 | |
| BHLH28 | | AT5G46830 | | | 3.84651 | | 0.00005 | | 0.000829337 | |
| SAMDC2 | | AT5G15950 | | | 2.98766 | | 0.00005 | | 0.000829337 | |
| TRY | | AT5G53200 | | | 2.44367 | | 0.0001 | | 0.00152628 | |
| LDOX | | AT4G22880 | | | 2.1626 | | 0.00005 | | 0.000829337 | |
| RGL2 | | AT3G03450 | | | 2.00591 | | 0.00005 | | 0.000829337 | |
| CCA1 | | AT2G46830 | | | 1.59851 | | 0.00005 | | 0.000829337 | |
| RD19A | | AT4G39090 | | | -0.906086 | | 0.00015 | | 0.00214411 | |
| DREB2C | | AT2G40340 | | | -1.03256 | | 0.00025 | | 0.0033076 | |
| BCB | | AT5G20230 | | | -2.75827 | | 0.00005 | | 0.000829337 | |
| TAT3 | | AT2G24850 | | | -2.85342 | | 0.00035 | | 0.00435491 | |
| PLIP2 | | AT1G02660 | | | -2.96077 | | 0.00005 | | 0.000829337 | |
| COL9 | | AT3G07650 | | | -3.07328 | | 0.00005 | | 0.000829337 | |
| FAMT | | AT3G44860 | | | -3.33914 | | 0.00005 | | 0.000829337 | |
| BT2 | | AT3G48360 | | | -3.72615 | | 0.00005 | | 0.000829337 | |
| **MYB25 Shoot: Jasmonic Acid Signaling** | | | | | | | | | | |
| **Gene** | **At #** | | | | **log2(fold_change)** | **p_value** | | | **q_value** | |
| MIP1A | AT3G21890 | | | | 5.46083 | 0.00115 | | | 0.0110262 | |
| BHLH28 | AT5G46830 | | | | 3.84651 | 0.00005 | | | 0.000829337 | |
| CLE12 | At1g68795 | | | | 3.0648 | 0.00295 | | | 0.0232388 | |
| PRE3 | At1g74500 | | | | 2.91781 | 0.007 | | | 0.0448004 | |
| LDOX | AT4G22880 | | | | 2.1626 | 0.00005 | | | 0.000829337 | |
| RGL2 | AT3G03450 | | | | 2.00591 | 0.00005 | | | 0.000829337 | |
| MYB29 | AT5G07690 | | | | 1.86067 | 0.00005 | | | 0.000829337 | |
| RD19A | AT4G39090 | | | | -0.906086 | 0.00015 | | | 0.00214411 | |
| GSTU4 | AT2G29460 | | | | -1.2851 | 0.00095 | | | 0.00950891 | |
| WRKY48 | At5g49520 | | | | -1.79781 | 0.00005 | | | 0.000829337 | |
| WRKY18 | AT4G31800 | | | | -2.90766 | 0.00005 | | | 0.000829337 | |
| JAZ10 | AT5G13220 | | | | -3.09429 | 0.00005 | | | 0.000829337 | |
| MIOX2 | AT2G19800 | | | | -3.32368 | 0.00005 | | | 0.000829337 | |
| BT2 | AT3G48360 | | | | -3.72615 | 0.00005 | | | 0.000829337 | |
| XTH22 | AT5G57560 | | | | -3.85728 | 0.00005 | | | 0.000829337 | |

| **MYB25 Shoot: Miscellaneous** | | | | |
| --- | --- | --- | --- | --- |
| **Gene** | **At #** | **log2(fold_change)** | **p_value** | **q_value** |
| SNRNP25 | At3g07860 | 4.42159 | 0.00715 | 0.0454163 |
| PER18 | At2g24800 | 2.5973 | 0.0021 | 0.0176838 |
| PSBZ | ATCG00300 | 2.36706 | 0.00005 | 0.000829337 |
| AMT1-2 | AT1G64780 | 2.16956 | 0.00005 | 0.000829337 |
| ATHB-52 | AT5G53980 | 2.04635 | 0.00005 | 0.000829337 |
| GRXC6 | At4g33040 | 2.03698 | 0.00005 | 0.000829337 |
| CWINV6 | AT5G11920 | 1.23299 | 0.0004 | 0.00482883 |
| CHI1 | At3g55120 | 0.82501 | 0.00015 | 0.00214411 |
| RAV1 | AT1G13260 | -0.573136 | 0.0065 | 0.0424038 |
| ERF054 | AT4G28140 | -2.26243 | 0.006 | 0.0400178 |
| TIFY11A | AT1G17380 | -2.28838 | 0.00005 | 0.000829337 |
| LEA29 | AT3G15670 | -2.53168 | 0.00015 | 0.00214411 |
| DREB1F | At1g12610 | -2.74327 | 0.00065 | 0.00712925 |
| TIFY 5B | AT2G34600 | -5.03223 | 0.00005 | 0.000829337 |
