# Supplementary material for: Characterization of Arabidopsis thaliana R2R3 S23 MYB Transcription Factors as Novel Targets of the Ubiquitin Proteasome-Pathway and Regulators of Salt Stress and Abscisic Acid Response
Source: Front Plant Sci. 2021 Aug 19;12:629208. doi: 10.3389/fpls.2021.629208 (PMC8417012; doi:10.3389/fpls.2021.629208)
Supplement: Supplementary file 9 [file Table_3.DOCX]

| **Supplementary Table 3 \| Complete list of primers used in this work** | |
| --- | --- |
| **Primers used for MYB1 cloning** | |
| MYB1-NdeI-FW | GGAGATATACATATGcatatgccATGGAGGCAGAGATCG |
| MYB1-RW-EcoRI | GGAGATATACATATGgaattcTTATGTGGACAGGAC |
| proMYB1FW1.6kb | GAATTTTGATACGTACGTGC |
| proMYB1gDNAGUSFW | GTTGAAAAGTCTCAATAAAGCTTagaattttgatacgtacgtgca |
| proMYB1gDNAGUSRW | tttctacaggacgtaacatcccgggTGTGGACAGGACATTGGTTATG |
| MYB1cDNAYFPFW | ggcgcgccactagtggatccATGGAGGCAGAGATCGT |
| MYB1cDNAYFPRW | cccttgctcaccatcccgggTGTGGACAGGACATTGGTTATG |
| **Primers used for MYB25 cloning** | |
| MYB25-FW | ATGAACGGAGAAATCTCTCGTCCGC |
| MYB25-RW-NoStop | GCTCAAAGCCCTTAAATC |
| MYB25-1.6-kb-Downstream | GTACATGTCAATAATGCTCTGGC |
| MYB25-RW-Upstream-ATG | CGGAAATAAAATTGAAATTTC |
| **Primers used for MYB109 cloning** | |
| MYB109-FW | ATGGAAGGAGAAACTCATC |
| MYB109-RW-NoStop | TGATCTAGGATTTTATA |
| MYB109-1.6-kb-Downstream | GTTAAGCAAAAGATTATATCC |
| MYB109-RW-Upstream-ATG | TTACACAAAAAAAGTTTCG |
| **Primers used for RT-PCR** | |
| pMDC-GFP6-FW | ATGAGTAAAGGAGAAGAACTTTTC |
| pMDC-GFP6-RW | TTATTTGTATAGTTCTACCATGC |
| DDB1a-1200-FW | GAATGTGGTCGTTGAAATC |
| DDB1a-1450-RW | GCCACTCGTCCCGCAAT |
| **Primers used for qRT-PCR** | |
| MYB25-qRT-RW | CACTTTACTCTTTCCGCCGC |
| MYB25-qRT-FW | CGGAGAAATCTCTCGTCCGC |
| Actin2-qRT-FW | CCTGCCATGTATGTTGCCATT |
| Actin 2-qRT-RW | AATCGAGCACAATACCGGTTGT |
| dPGM-qRT-FW | AACGACCTCGAGATTCCGGG |
| dPGM-qRT-RW | CCGGCAGAGGAAGCTTGTGT |
| CYP71A13-qRT-FW | AACGATAAAGCGGATTTCGTGGAT |
| CYP71A13-qRT-RW | TCGAAGTTGTTGACGTTCCTCC |
| NIG1-qRT-FW | AAACCGGCGCATGGTAGAGA |
| NIG1-qRT-RW | AAACCGCGTCTTCGAGCAAC |
| AT3G21352-qRT-FW | TCTAGGAGGTGGAGGGCGTAA |
| AT3G21352-qRT-RW | CTGGACAACGACATGGGCAG |
